# Using next-generation DNA sequence data for genetic association tests based on allele counts with and without consideration of zero inflation

**DOI:** 10.1186/s12919-016-0062-5

**Published:** 2016-10-18

**Authors:** Rosa González Silos, Özge Karadag, Barbara Peil, Christine Fischer, Maria Kabisch, Carine Legrand, Justo Lorenzo Bermejo

**Affiliations:** 1 Institute of Medical Biometry and Informatics, University of Heidelberg, Heidelberg, 69120 Germany; 2 Department of Statistics, Hacettepe University, Ankara, 06800 Turkey; 3 Institute of Human Genetics, University of Heidelberg, Heidelberg, 69120 Germany; 4 Molecular Genetics of Breast Cancer (B072), German Cancer Research Center (DKFZ), Heidelberg, 69120 Germany

## Abstract

The relationship between genetic variability and individual phenotypes is usually investigated by testing for association relying on called genotypes. Allele counts obtained from next-generation sequence data could be used for this purpose too. Genetic association can be examined by treating alternative allele counts (AACs) as the response variable in negative binomial regression. AACs from sequence data often contain an excess of zeros, thus motivating the use of Hurdle and zero-inflated models. Here we examine rough type I error rates and the ability to pick out variants with small probability values for 7 different testing approaches that incorporate AACs as an explanatory or as a response variable. Model comparisons relied on chromosome 3 DNA sequence data from 407 Hispanic participants in the Type 2 Diabetes Genetic Exploration by Next-generation sequencing in Ethnic Samples (T2D-GENES) project 1 with complete information on diastolic blood pressure and related medication. Our results suggest that in the investigation of the relationship between AAC as response variable and individual phenotypes as explanatory variable, Hurdle-negative binomial regression has some advantages. This model showed a good ability to discriminate strongly associated variants and controlled overall type I error rates. However, probability values from Hurdle-negative binomial regression were not obtained for approximately 25 % of the investigated variants because of convergence problems, and the mass of the probability value distribution was concentrated around 1.

## Background

Diastolic blood pressure (DBP) measures the pressure during heart relaxation. High DBP increases the risk of heart attacks, stroke, and kidney failure. DBP is dependent on the age, gender, and medication of an individual [1]. A genetic component has been identified for high DBP [2].

The relationship between genetic variability and DBP is commonly investigated by testing for association between called genotypes and phenotypes. In sequence data, genotypes are called relying on sequence reads. Alternatively, association tests without calling genotypes have been suggested [3].

Depth in DNA sequence data refers to the number of reads at a position. In next-generation sequence data there are 2 counts of allelic depth, the number of reference alleles, and the number of alternative alleles. Here we use the number of alternative allele counts (AACs) and the total read depth. AACs are genotype measurements that are more informative than called genotypes in the sense that the 2 counts—“zero reference alleles out of 100” and “1 reference allele out of 100”—would translate into the same called genotype. In other words, after applying user-defined data quality filters, the uncertainty in genotype calling is rarely taken into account in statistical analyses.

Several statistical procedures can be used for testing association between allele counts and a phenotype of interest. When AAC is regarded as the response variable and the total read depth as an offset, standard models for count data can be used. The response variable can also be defined as the ratio of the AAC to the total read depth. In this case, linear regression can be used to investigate the genotype–phenotype relationship. It is also possible to model the phenotype as a response variable, and the ratio “AAC/total read depth” as an explanatory variable.

We used data from the Genetic Analysis Workshop (GAW) 19, which are provided for 1943 Hispanic samples that have been whole-exome sequenced as part of the Type 2 Diabetes Genetic Exploration by Next-generation sequencing in Ethnic Samples (T2D-GENES) Project 1 to compare different regression techniques.

## Methods

Phenotype data were provided for 1943 Hispanics who participated in the T2D-GENES Project 1, and included 4 variables: DBP, age, gender, and medication [4, 5]. We excluded individuals with missing information on medication; that is, the investigated data set included 407 patients. To be able to treat DBP both as response and as explanatory variable, we regressed the measured DBP on age, sex, and medication, and used the residuals as a new phenotype variable that we denominated “adjusted-DBP.”

Regarding genotype measurements, exonic regions were isolated using Agilent TruSeq capture reagents, and individually barcoded samples sequenced in Illumina HiSeq 2000 instruments. Across the coding sequence of 18,281 genes, an average read depth of approximately 82-fold was reached. We used VCFtools (version 0.1.4a) to extract the data needed: counts of reference and alternative alleles (AD field in the FORMAT tag of the variant call format [vcf] file), genotype (GT field in the FORMAT tag), and average genotype quality (GQ field in the FORMAT tag). Total read depth was calculated as the sum of the counts for the reference and the alternative alleles. We also used VCFtools to calculate the minor allele frequency (MAF). Our analysis focused on biallelic variants in chromosome 3. Variants with a MAF of less than 0.003 were excluded, leaving a total of 8957 variants for analysis.

We compared different regression models and treated AAC both as response and as explanatory variable. The relationship between AAC as response variable (total read depth was used as offset), and adjusted-DBP as explanatory variable was first investigated by negative binomial regression. Zero-inflated negative binomial (ZI-NB) and Hurdle-negative binomial (Hu-NB) models are more flexible than negative binomial regression in the presence of zero inflation [6, 7]. Comparisons between the complete model with adjusted-DBP, and a null model with only the intercept relied on the deviance (minus twice the difference of log null and complete model likelihoods) investigated with a chi-squared test.

We also investigated the genotype–phenotype relationship based on the ratio “AAC/total read depth” and adjusted-DBP, as response and explanatory variables in standard linear and robust linear regression [8].

Type I error rates were roughly estimated assuming that the large majority of the 8957 investigated variants were under the null hypothesis of no genetic association. Quantile–quantile (Q-Q) plots were used to explore possible disparities between small probability values (*p* values) from different regression models. These analyses do not amount to a systematic model comparison relying on extensive simulations, but they could be suggestive of the most promising modeling approaches.

R-packages stats, pscl, and robust were used to fit negative binomial/linear regression, ZI-NB/Hu-NB, and robust linear regression models, respectively.

## Results

**Fig. 1:**
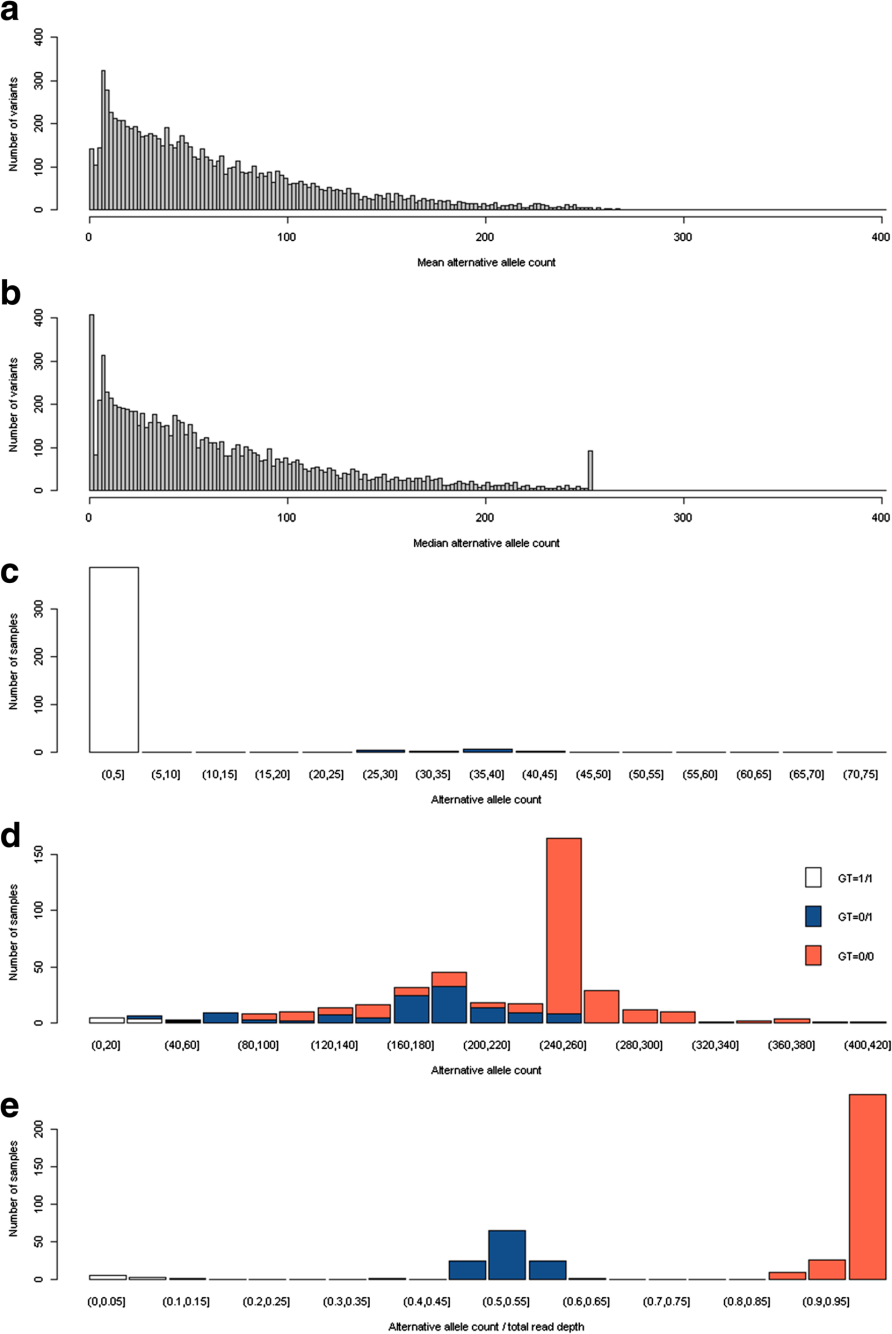
Distribution of AAC. **a** Mean AACs per variant. **b** Median AACs per variant. **c** Exemplary zero-inflated AACs distribution grouped by genotype for the variant in position Chr3:391100 with MAF equal to 0.0258. **d** Exemplary skewed AACs distribution grouped by genotype for the variant in position Chr3:16249998 with MAF equal to 0.1683. **e** Exemplary comparison of the ratios (AAC/total read depth) and called genotypes for the variant in position Chr3:16249998

Figure 1 shows the distribution of AACs for all 8957 investigated variants. Panel A shows the distribution of mean AACs; panel B shows the distribution of median AACs; and panel C, which represents the AAC distribution variant in position Ch3:391100, illustrates the frequent large proportion of zero counts. Noteworthy is that panel B revealed 105 variants with a median AAC exactly equal to 254. This peak was not apparent when mean AACs were represented. To investigate the possible origin of this peak, we plotted a histogram of AACs for the variant in position Ch3:16249998, which presented a median (mean) AAC of 254 (40.03). This variant showed a MAF of 0.168 (panel D: 280 reference allele homozygotes, 117 heterozygotes, and 10 alternative allele homozygotes). The presence of 35 variants with a median AAC greater than 254 in panel B, combined with panel (D), suggest that downsampling, if present, was incomplete. A field with information on the possible downsampling was not available. Panel E compares the distribution of the “AAC/total read depth” ratio with the distribution of called genotypes.

Table 1 shows rough type I error rates for the 8957 investigated variants in chromosome 3. For example, the use of negative binomial regression to investigate the relationship between AAC as response variable, and adjusted-DBP as explanatory variable, resulted in 8871 nonmissing *p* values, 39 % of which were smaller than 0.05. No convergence was the principal cause of missing *p* values and increasing the number of iterations did not alleviate the problem. Consideration of zero inflation in negative binomial regression increased the percentage of missing *p* values, which amounted to 26 % for both ZI-NB and Hu-NB. The proportion of nonmissing *p* values less than 0.05 was 6.4 % for ZI-NB and 3.5 % for Hu-NB regression models.

**Table 1:** Overall type I error rates

Response variable	Explanatory variable	Regression model	# *p* values	# Nonmissing *p* values	% Nonmissing *p* values	% Nonmissing *p* values under 0.05
AAC	Adjusted DBP	Negative binomial (NB)	8957	8871	99.0	0.388
		Zero-inflated NB	8957	6608	73.8	0.064
		Hurdle NB	8957	6585	73.5	0.035
AAC/total read depth	Adjusted DBP	Standard linear	8957	8947	99.9	0.062
		Robust linear	8957	1509	16.8	0.068
Adjusted DBP	AAC/total read depth	Standard linear	8957	8947	99.9	0.062
		Robust linear	8957	8917	99.6	0.127

**Fig. 2:**
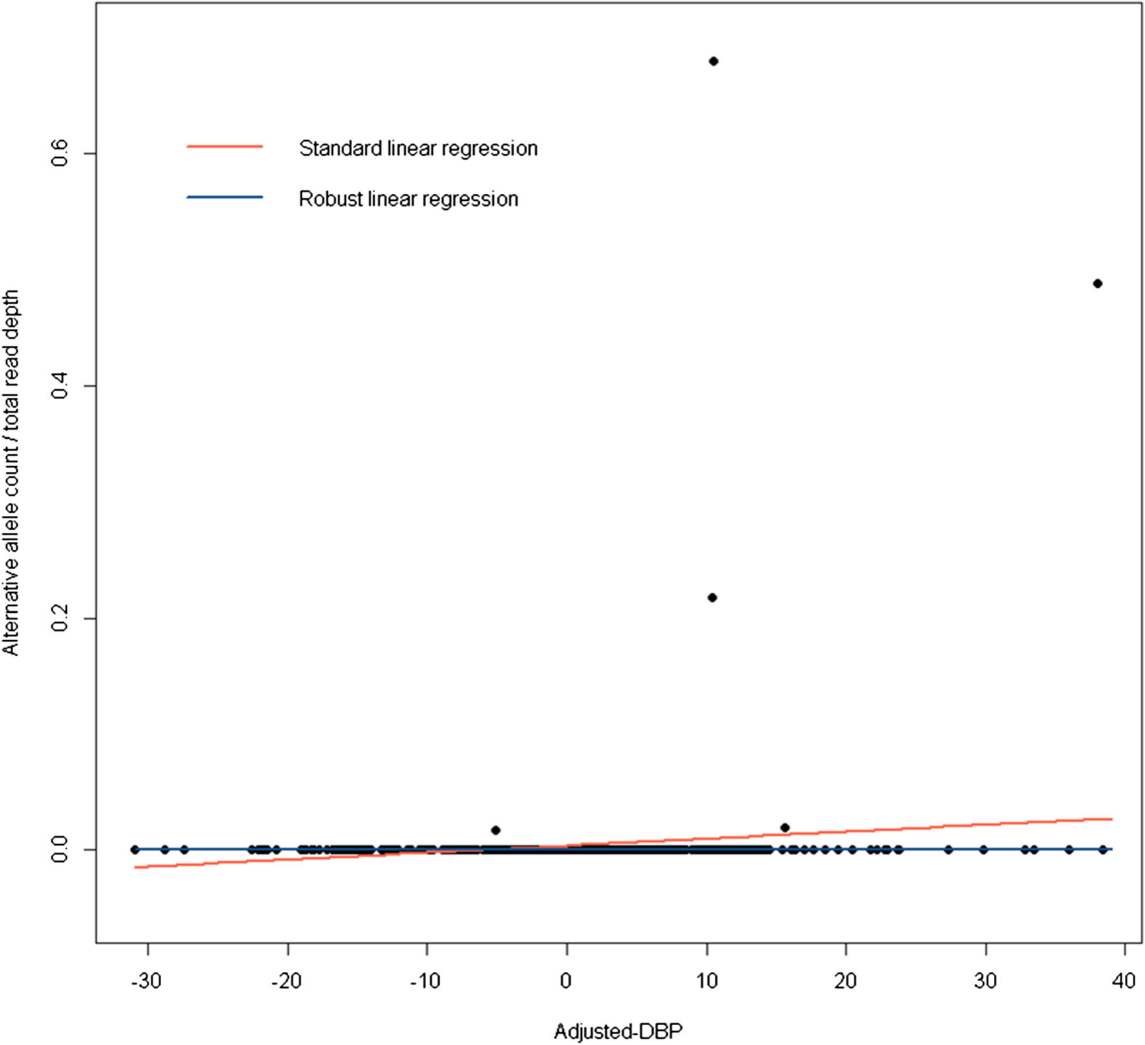
Scatter graph and fitted model for the variant in position Chr3:361487

Among the 7 investigated models, standard linear regression generated the lowest proportion of missing *p* values (approximately 1 per 1000 tested variants). Note that treating the ratio “AAC/total read depth” as a response or explanatory variable in standard linear regression resulted in identical *p* values. In robust linear regression, treating the ratio “AAC/total read depth” as a response variable resulted in a high proportion of missing *p* values (83 %). The reason was a perfect model fit after downweighting outliers (observations with a large “AAC/total read depth” ratio) by robust regression. Figure 2 illustrates this problem.

Motivated by this result, the rest of the manuscript focuses on the 4 regression models with the best calibrated overall rough type I error rates: ZI-NB and Hu-NB, standard linear regression, and robust linear regression with the ratio “AAC/total read depth” as a response variable. Table 2 shows rough type I error rates stratified by 3 characteristics of the variants: median AAC, MAF, and the average genotype quality. Only 1.6 % of the variants presented a median AAC equal to or larger than 254, but this cutoff was chosen based on the observed peak in Fig. 1, panel b. The proportion of *p* values of less than 0.05 from ZI-NB and Hu-NB regression models was lower for variants with a median AAC of less than 60 than for variants with higher median AACs. By contrast, this proportion did not depend on the median AAC for standard and robust linear regression.

**Table 2:** Type I error rates stratified by variant-specific AAC, MAF, and average genotype quality

Response variable	Regression model	Median AAC	# *P* values	# Nonmissing *p* values	% Nonmissing *p* values	% Nonmissing *p* values under 0.05
AAC	ZI-NB	[0,60]	4728	3598	76.1	0.050
		(60,254)	4089	2917	71.3	0.080
		254	105	84	80.0	0.083
		(254,426]	35	9	25.7	0.000
	Hu-NB	[0,60]	4728	3580	75.7	0.029
		(60,254)	4089	2912	71.2	0.043
		254	105	84	80.0	0.036
		(254,426]	35	9	25.7	0.111
AAC/total read depth	Standard linear (SL)	[0,60]	4728	4719	99.8	0.062
		(60,254)	4089	4088	100.0	0.062
		254	105	105	100.0	0.029
		(254,426]	35	35	100.0	0.114
	Robust linear (RL)	[0,60]	4728	767	16.2	0.068
		(60,254)	4089	708	17.3	0.068
		254	105	10	9.5	0.100
		(254,426]	35	24	68.6	0.083
Response variable	Regression model	MAF	# *P* values	# Nonmissing *p* values	% Nonmissing *p* values	% Nonmissing *p* values under 0.05
AAC	ZI-NB	[0.003,0.01]	3707	3694	99.6	0.036
		(0.01,0.05]	1927	1895	98.3	0.144
		>0.05	3323	1019	30.7	0.016
	Hu-NB	[0.003,0.01]	3707	3695	99.7	0.044
		(0.01,0.05]	1927	1886	97.9	0.031
		>0.05	3323	1004	30.2	0.011
AAC/total read depth	SL	[0.003,0.01]	3707	3698	99.8	0.058
		(0.01,0.05]	1927	1927	100.0	0.053
		>0.05	3323	3322	100.0	0.072
	RL	[0.003,0.01]	3707	2	0.1	0.000
		(0.01,0.05]	1927	0	0.0	0.000
		>0.05	3323	1507	45.4	0.068
Response variable	Regression model	Average genotype quality	# *P* values	# Nonmissing *p* values	% Nonmissing *p* values	% Nonmissing *p* values under 0.05
AAC	ZI-NB	Low	2985	2973	99.6	0.067
		Medium	2986	2810	94.1	0.060
		High	2986	825	27.6	0.058
	Hu-NB	Low	2985	2974	99.6	0.044
		Medium	2986	2812	94.1	0.031
		High	2986	799	26.7	0.016
AAC/total read depth	SL	Low	2985	2975	99.7	0.056
		Medium	2986	2986	100.0	0.062
		High	2986	2986	100.0	0.068
	RL	Low	2985	9	0.3	0.000
		Medium	2986	96	3.2	0.031
		High	2986	1404	47.0	0.071

Interestingly, convergence problems and missing *p* values from ZI-NB and Hu-NB regression were particularly relevant for variants with a MAF greater than 0.05. The relationship between rough type I error rates and the MAF showed no clear pattern. For example, the proportion of *p* values less than 0.05 from Hu-NB regression decreased with increasing the MAF, and showed a U-pattern for standard linear regression. As expected, *p* values from robust linear regression were practically not available for variants with a MAF of less than 0.05.

Variants were also grouped into 3 balanced categories according to their average genotype qualities (low: GQ field in the vcf file from 30 to 60,000; medium: GQ from 60,000 to 210,000; and high: GQ >210,000). Rough type I error rates slightly decreased with increasing average genotype quality for ZI-NB and Hu-NB regression models. By contrast, approximate type I error rates increased with increasing average genotype quality for standard and for robust linear regression. *P* values from robust linear regression were practically not available for variants with an average genotype quality of less than 60,000.

**Fig. 3:**
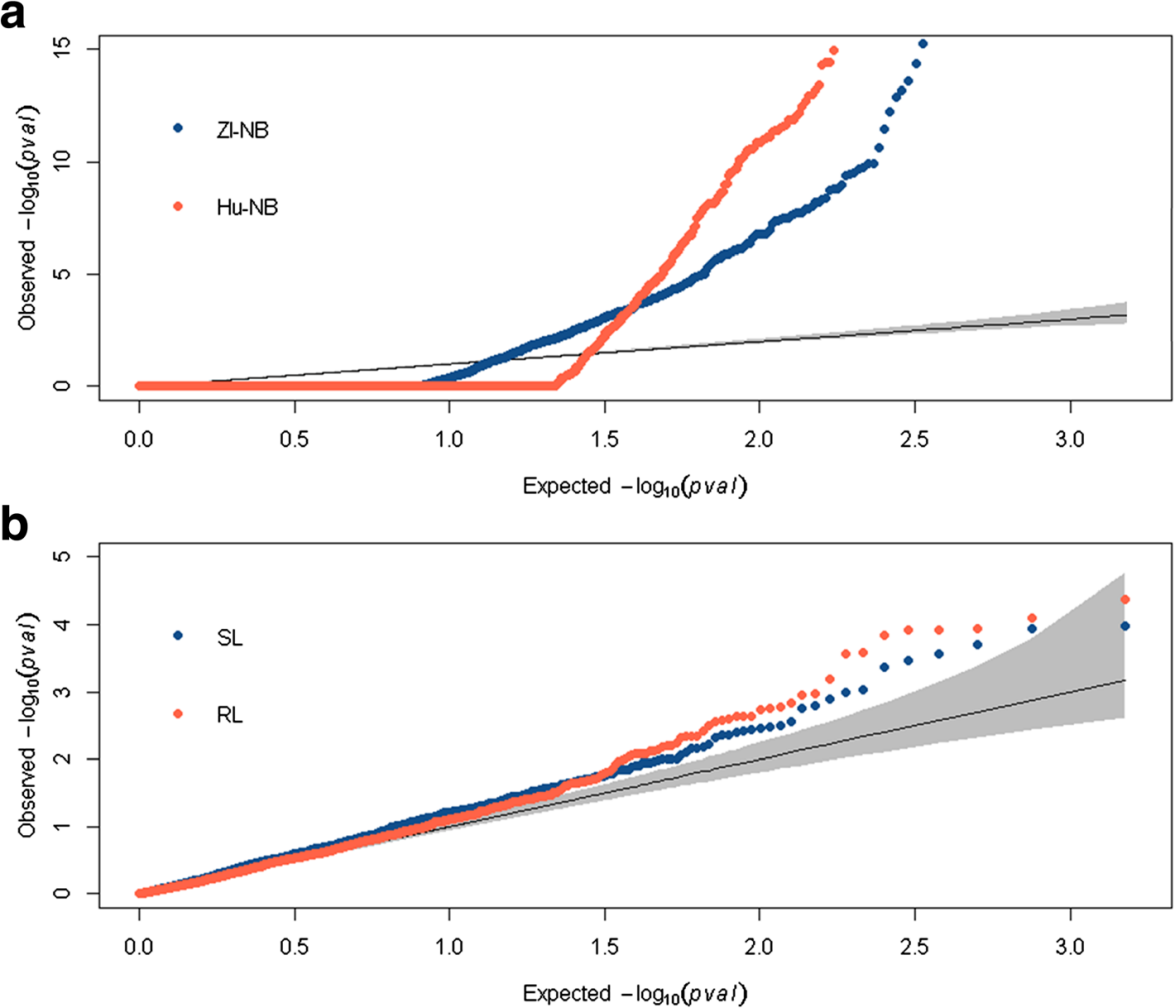
QQ plots for ZI-NB and Hu-NB models treating the AACs as a response variable (**a**), and for standard and robust linear regression models treating the ratio (AAC/total read depth) as a response variable (**b**). Gray shape represents the 95 % confidence bands regions

Figure 3 investigates the distributions of *p* values for the 4 most promising regression models. To interpret results, the proportion of missing *p* values and the properties of each model discussed above should be kept in mind. Although 4 % of *p* values were smaller than 0.05 for Hu-NB and 6 % for ZI-NB regression, the mass of the *p* value distributions was concentrated around 1 (87 % of the *p* values from the Hu-NB and 80 % of the *p* values from the ZI-NB model were higher than 0.95). Hu-NB regression picked out variants with small *p* values more clearly than the ZI-NB version (A) with similar proportions of missing *p* values, and similar dependence patterns of the rough type I error rates on the median AACs and on the average genotype quality. However, test statistics from ZI-NB regression were likely not properly calibrated for variants with MAFs in the interval (0.01, 0.05] (see Table 2 in which 14 % of the *p* values were less than 0.05).

Figure 3b compares the standard and robust versions of linear regression. The robust model, somewhat more clearly, picked out variants with small *p* values, but *p* values were within 95 % confidence bands. It should be kept in mind that robust linear regression only generated *p* values for variants with a MAF greater than 0.05, and for variants with an average genotype quality greater than 60,000. Note the differential scales of the *y*-axis in Fig. 3a and b. Based on these preliminary results, which should be complemented with simulations in the future, Hu-NB regression seems to be a good candidate to investigate the relationship between AAC as a response variable, and individual phenotypes as an explanatory variable.

## Discussion

The direct inspection of raw counts is an established approach to investigate differential expression based on RNA sequence data (see, eg, the R-package DESeq2), but the analysis of DNA sequence data almost always focuses on called genotypes. Satten et al [3] proposed using the proportion of calls for the minor allele instead of called genotypes. This proposal motivated the present investigation, where we used the ratio “AAC/total read depth.” A large proportion of false-positive associations based on sequence data was reported in earlier versions of the GAW [9]. Recommendations to address this problem usually rely on called genotypes; for example, accounting for population substructure and conducting gene-based or collapsing association tests.

The possibility of using AAC instead of called genotypes motivated the present research. The underlying distribution of AAC reflects the reliability of called genotypes, and a direct analysis of AAC would permit integration of this uncertainty into association results. However, next-generation sequence data is noisy and the quality of the called genotypes depends on the chromosomal position. Lots of variants are artifactual and rely on small counts.

The exclusive use of real data without additional simulations is a limitation of the present study. Nonparametric and quintile regression could be potential alternatives to robust linear regression in the presence of outlying AACs. We investigated the dependency of results on AAC, but data was not available for variants with low-quality genotype calls. It would be interesting to examine the behavior of Hu-NB regression for these variants.

## Conclusions

We took advantage of real data to compare different models that investigate the relationship between sequence allele counts and the adjusted-DBP. The methods with the best control of rough type I error rates were ZI-NB and Hu-NB regression for the relationship between AAC and adjusted-DBP, and standard and robust linear regression for the relationship between the ratio “AAC/total read depth” as a response variable and adjusted-DBP as an explanatory variable.

The simultaneous consideration of discriminative ability of variants with small *p* values, occurrence of convergence problems, and robustness of *p* values against departing DBP observations indicated that Hu-NB regression constitutes a promising approach to assess the association between AAC and individual phenotypes.
